# Tumor suppressor miR-1 inhibits tumor growth and metastasis by simultaneously targeting multiple genes

**DOI:** 10.18632/oncotarget.14927

**Published:** 2017-01-31

**Authors:** Cuilian Liu, Song Zhang, Qizhi Wang, Xiaobo Zhang

**Affiliations:** ^1^ College of Life Sciences and Laboratory for Marine Biology and Biotechnology of Qingdao National Laboratory for Marine Science and Technology, Zhejiang University, Hangzhou 310058, The People's Republic of China; ^2^ Department of Gastroenterology, The First Affiliated Hospital of Bengbu Medical College, Bengbu 233030, The People's Republic of China

**Keywords:** miR-1, target gene, tumor growth, metastasis

## Abstract

Cancer progression depends on tumor growth and metastasis, which are activated or suppressed by multiple genes. An individual microRNA may target multiple genes, suggesting that a miRNA may suppress tumor growth and metastasis via simultaneously targeting different genes. However, thus far, this issue has not been explored. In the present study, the findings showed that miR-1 could simultaneously inhibit tumor growth and metastasis of gastric and breast cancers by targeting multiple genes. The results indicated that miR-1 was significantly downregulated in cancer tissues compared with normal tissues. The miR-1 overexpression led to cell cycle arrest in the G1 phase in gastric and breast cancer cells but not in normal cells. Furthermore, the miR-1 overexpression significantly inhibited the metastasis of gastric and breast cancer cells. An analysis of the underlying mechanism revealed that the simultaneous inhibition of tumor growth and metastasis mediated by miR-1 was due to the synchronous targeting of 6 miR-1 target genes encoding cyclin dependent kinase 4, twinfilin actin binding protein 1, calponin 3, coronin 1C, WAS protein family member 2 and thymosin beta 4, X-linked. *In vivo* assays demonstrated that miR-1 efficiently inhibited tumor growth and metastasis of gastric and breast cancers in nude mice. Therefore, our study contributed novel insights into the miR-1′s roles in tumorigenesis of gastric and breast cancers.

## INTRODUCTION

Cancer is the most common cause of disease-related death in humans worldwide, with more than 10 million new cases every year [1]. Tumor development and progression is a multistep process, in which cells progress from benign to malignant tumors. Currently, six hallmarks of cancers have been proposed to constitute an organizing principle that provides a solid foundation for the understanding of cancer biology [2]. The six hallmarks of cancers can enable tumor growth and metastatic dissemination. Therefore, the inhibition of tumor growth and metastatic dissemination is the primary challenge of cancer therapy. Standard cancer treatments currently include surgical intervention, radiation and chemotherapeutic drugs. However, radiation and chemotherapeutic drugs can kill normal cells and cause toxicity in patients. In recent years, many chemotherapeutic drugs targeting cancer-related molecules have been discovered and tested in clinical trials due to an improved understanding of tumor biology. ZSKT474, a PI3K inhibitor, suppresses tumor growth by inducing G0/G1 arrest and is in a phase I clinical trial for the treatment of solid tumors [3]. Epidermal growth factor receptor (EGFR)-targeted monoclonal antibodies (mAbs), such as panitumumab and cetuximab, have had an impact on metastatic colorectal cancer therapy [4]. The RAS proteins, key regulators of malignant transformation, are aberrantly expressed in most human tumors. Rational therapies that target the RAS pathways may inhibit tumor growth, survival and metastasis [5, 6]. Tumor development and metastasis depend on the regulation of gene expression. As novel cancer therapeutic agents, microRNAs (miRNAs) have attracted an increasing amount of attention for their capacity to activate or suppress tumor-associated gene expression.

miRNAs are a group of small noncoding RNAs that suppress gene expression by base pairing with 3′-untranslated region (3′-UTR) of the target mRNAs, resulting in mRNA degradation or translation inhibition [7]. Thus far, many reports have identified miRNAs roles in various cellular processes, including differentiation, apoptosis, cell proliferation, metabolism, immunity, and development. Abnormal miRNAs expression is associated with different pathological conditions, such as cardiovascular and neuronal disorders, inflammation, and cancer [8]. Experimental and clinical studies have demonstrated that aberrations in miRNAs expression are associated with tumorigenesis and cancer metastasis. miR-148b, which is frequently down-regulated in gastric cancer, can suppress gastric cancer cell growth by targeting the cholecystokinin-B receptor [9]. miR-200 is shown to inhibit cancer cell migration by directly targeting the transcription factors ZEB1 and ZEB2 [10]. The forced expression of miR-200 abrogates the capacity of tumor cells to invade and metastasize from primary tumors [11]. In most human malignancies, miR-21 acts as an oncogene by silencing the expression of many tumor suppressor genes related to proliferation, apoptosis and invasion [12]. However, the majority of studies on miRNAs have focused on one miRNA and one target gene. In fact, miRNAs exhibit enormous regulatory potential, because a single miRNA is generally believed to target multiple mRNAs [13, 14]. For example, miRNAs from the miR-16 family induce G0/G1 cell cycle arrest by silencing multiple cell cycle genes simultaneously, including *Cyclin D1*, *Cyclin D3*, *Cyclin E1* and *CDK6*, rather than an individual target [15]. However, no reports have examined whether an individual miRNA can simultaneously function in tumorigenesis and cancer metastasis by regulating different target genes. Therefore, we aimed to identify a multifunctional miRNA that can suppress both tumor growth and metastasis by synchronously targeting multiple genes.

To address this issue, miR-1 was characterized in this study. The results indicated that miR-1 was differentially expressed in both cancerous and normal cells. Further analysis showed that miR-1 simultaneously targeted *CDK4* (cyclin-dependent kinase 4), *TWF1* (twinfilin actin binding protein 1), *WASF2* (WAS protein family, member 2), *CNN3* (calponin 3, acidic), *CORO1C* (coronin, actin binding protein, 1C) and *TMSB4X* (thymosin beta 4, X-linked), key genes involved in the cell cycle and metastasis, leading to the simultaneous inhibition of tumor growth and metastasis.

## RESULTS

### Downregulation of miR-1 in cancer cells and gastric cancer tissues

**Figure 1:**
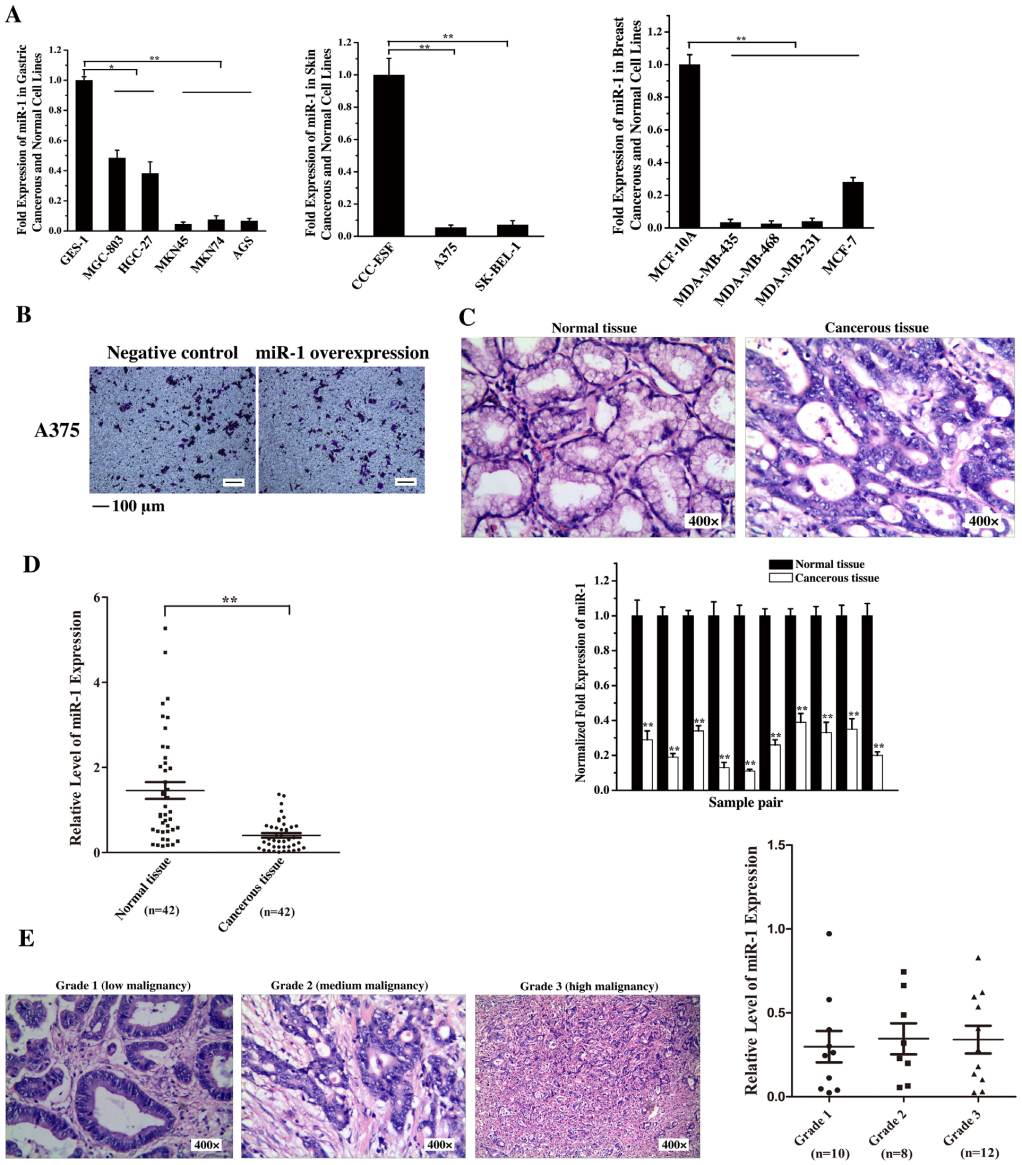
Downregulation of miR-1 in gastric cancer cells and tissues **A**. The expression of miR-1 in gastric cancer, skin cancer, breast cancer and normal cell lines. miR-1 expression was measured by quantitative real-time PCR in cancer cells and compared with that in the normal GES-1, CCC-ESF and MCF-10A cells. **B**. Influence of miR-1 overexpression on human skin cancer A375 cell migration. A375 cells were transfected with the miR-1 precursor or the negative control. At 48 h after transfection, cell migration was examined. Representative images are shown. Scale bar, 100 μm. **C**. The expression of miR-1 in tumor specimens from gastric cancer patients. Cancerous tissue and corresponding normal tissue from the same patients were examined as paired samples (n=10). The samples were characterized using haematoxylin and eosin staining (400×) and quantitative real-time PCR of miR-1. **D**. Scatter plot showing the expression level of miR-1 in tumor (n=44) and corresponding normal samples (n=42) from gastric cancer patients. The expression of miR-1 was measured using quantitative real-time PCR. **E**. The expression of miR-1 in gastric cancers at various stages of differentiation. Cancer tissue samples were divided into three grades using hematoxylin and eosin staining (400×). The expression level of miR-1 in grade 1 (n=10), grade 2 (n=8) and grade 3 (n=12) samples was analyzed by quantitative real-time PCR. Statistically significant differences are indicated with asterisks (*, *p* < 0.05; ** *p* < 0.01).

To reveal the role of miR-1 in tumorigenesis, the expression levels of miR-1 in the cells of skin cancer, breast cancer and gastric cancer, three of the most common malignant cancers worldwide, were examined. The quantitative real-time PCR results showed that the miR-1 expression was significantly decreased in all cancer cells compared with that in the corresponding normal cells (Figure 1A), indicating that miR-1 might be a tumor suppressor. The cancer cell metastasis analysis revealed that the miR-1 overexpression in human skin cancer A375 cells had no effect the cancer cell migration compared with the control (Figure 1B). Thus human skin cancer A375 cells were not included in the following assays.

To further characterize the differential expression of miR-1 in gastric cancerous and normal cells, the primary tumor specimens from 10 patients with gastric cancer were assayed. The results showed that the miR-1 expression level in cancerous tissues was significantly lower than that in the paired normal tissues (Figure 1C). To evaluate the miR-1 expression in more clinical samples, 42 pairs of cancerous tissues and corresponding normal tissues from the same patients with gastric cancer were examined. The results indicated that there was a significant correlation between miR-1 expression level and tumorigenesis (Figure 1D).

Based on the degree of tumor cell differentiation detected histopathologically, the gastric primary tumors were classified into three grades, i.e., grade 1, 2 or 3. The data presented that the expression level of miR-1 was not correlated with tumor cell differentiation (Figure 1E), indicating that the miR-1 expression was downregulated in gastric cancers at various stages of differentiation.

Taken together, these findings revealed a significant correlation between miR-1 downregulation and primary human tumorigenesis.

### Inhibition of gastric and breast cancer cell growth by miR-1

**Figure 2:**
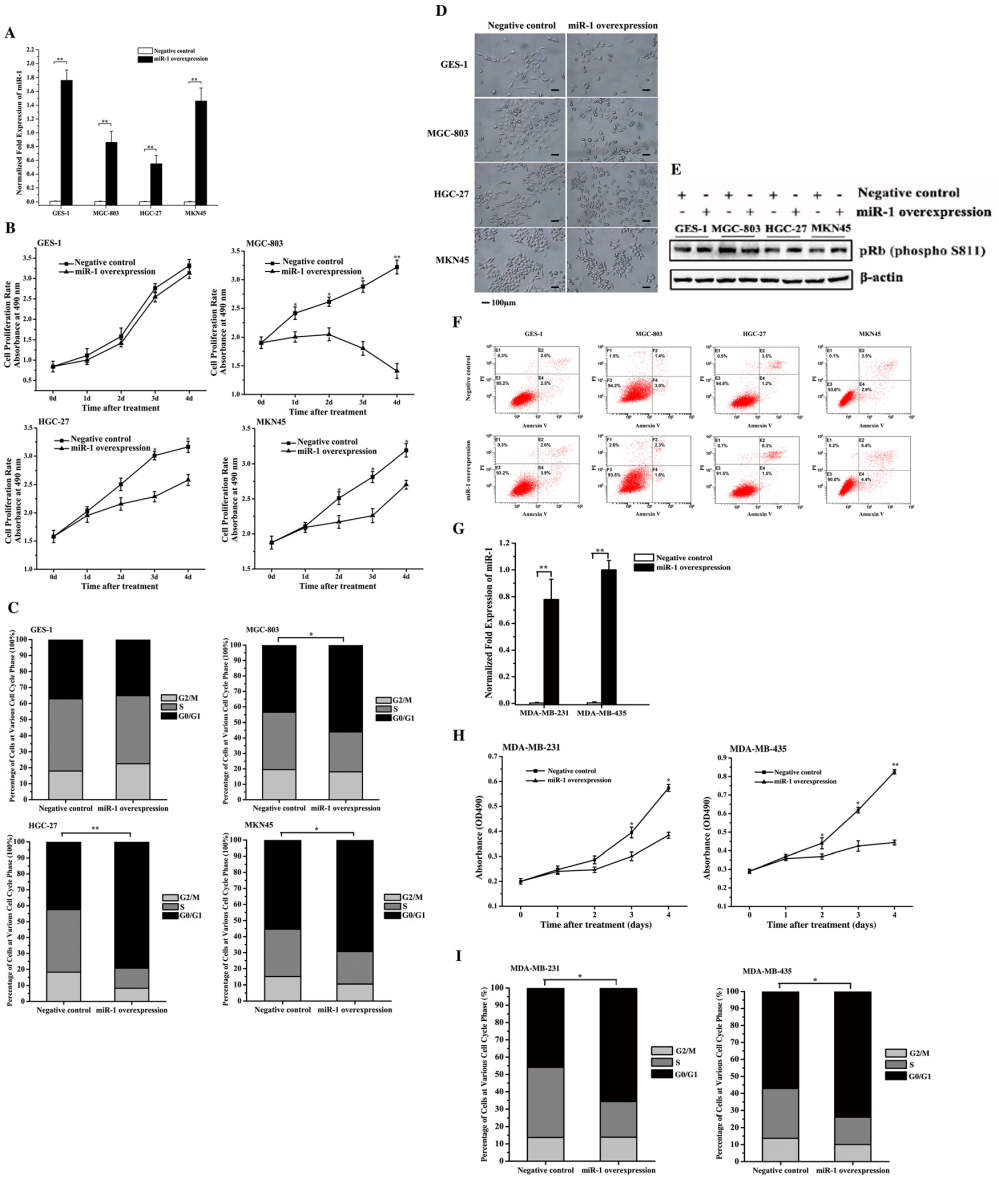
Effects of miR-1 on the growth of gastric and breast cancer cells and normal cells **A**. miR-1 overexpression in gastric cancer and normal cells. MGC-803, HGC-27, MKN45 and GES-1 cells were transfected with the miR-1 precursor or the negative control miRNA. At 48 h after transfection, the miR-1 expression was detected with quantitative real-time PCR. **B**. Cell proliferation rate. Cells were transfected with the precursor miR-1 or the negative control miRNA. From 1 d to 5 d after transfection, cell growth was examined using cell proliferation assays. **C**. Effects of miR-1 on the cell cycle. At 48 h after cells were transfected with the miR-1 precursor or the negative control, the cell cycle of cancer and normal cells was evaluated with flow cytometry. **D**. Cell senescence analysis. Cells transfected with the precursor miR-1 or the negative control miRNA were treated using senescence-associated β-galactosidase staining. Representative images are shown. Scale bar, 100 μm. **E**. Detection of pRb phosphorylation. Cells treated with the precursor miR-1 or the negative control miRNA were subjected to Western blot analysis with the phosphorylated pRb antibody (P-pRb). β-Actin was used as a control. **F**. Detection of gastric cancer cell apoptosis using flow cytometry. Cells transfected with the precursor miR-1 or the negative control miRNA were subjected to apoptosis assays with AnnexinV. **G**. The overexpression of miR-1 in breast cancer cells. Breast cancer cells (MDA-MB-231 and MDA-MB-435) were transfected with 30 nM of the miR-1 precursor or the negative control miRNA. After 48 h after, the cells were analyzed using quantitative real-time RT-PCR. **H**. The effect of miR-1 overexpression on breast cancer cell proliferation. Breast cancer cells were transfected with 30 nM of the miR-1 precursor or the negative control miRNA. Then, cell growth was examined daily. **I**. Effects of miR-1 overexpression on breast cancer cell cycle. The breast cancer cells transfected with the miR-1 precursor were subjected to cell cycle assays. In all panels, data are shown as mean ± standard deviation from three independent experiments (* *p* < 0.05; ** *p* < 0.01).

To investigate the role of miR-1 in cancer cell growth, miR-1 was overexpressed in gastric cancer cells (MGC-803, HGC-27 and MKN45) and normal gastric cells (GES-1) (Figure 2A). The results showed that the overexpression of miR-1 significantly inhibited the proliferation rates of gastric cancer cells compared with the negative control, while miR-1 overexpression had no effect on the growth of normal cells (Figure 2B). This inhibition of cancer cell proliferations by miR-1 suggested the involvement of miR-1 in the cell cycle or/and cancer cell senescence.

To elucidate the mechanism underlying the miR-1-mediated inhibition of gastric cancer cell growth, the cell cycle was analyzed in cells transfected with the precursor miR-1 and the negative control miRNA. The FACS analysis showed that the numbers of cells at G0/G1 phase in the miR-1-overexpressing HGC-27, MGC-803 and MKN45 cells were significantly increased compared with the controls (Figure 2C). However, miR-1 overexpression had no effect on the cell cycle of GES-1 cells (Figure 2C). These data indicated that miR-1 could initiate cell cycle arrest in the G0/G1 phase in cancer cells but not normal cells, contributing to the inhibition of cancer cell proliferation by miR-1.

To evaluate the effects of miR-1 on cell senescence, MGC-803, HGC-27, MKN45 and GES-1 cells were transfected with the miR-1 precursor, and were then examined for senescence. The senescence-associated β-galactosidase staining results revealed that not all cells were stained, indicating that senescence did not occur in the miR-1-overexpressing cancer and normal cells (Figure 2D). Moreover, the analysis of the pRb the phosphorylation, in which decreased phosphorylation is a marker of cell senescence [11], revealed that pRb was phosphorylated in all cells (Figure 2E), indicating that miR-1 did not affect gastric cancer cell senescence. The apoptosis assays demonstrated that percentages of apoptotic miR-1-overexpressing cancer cells were similar to those of the controls (Figure 2F), indicating that miR-1 had no effect on the apoptosis of gastric cancer cells.

The above data revealed that the miR-1-mediated inhibition of gastric cancer cell proliferation but not normal cell proliferation was due to cancer cell cycle arrest in G0/G1 phase.

To evaluate the role of miR-1 in the regulation of the cell cycle of breast cancer cells, miR-1 was overexpressed in MDA-MB-231 and MDA-MB-435 breast cancer cells (Figure 2G). The cell proliferation assay results indicated that miR-1 overexpression inhibited breast cancer cell growth compared with the control (Figure 2H). In addition, the cell cycle analysis showed that the number of cells in the G0/G1 phase was 20% higher after transfection with the miR-1 precursor than with the negative control (Figure 2I), indicating that miR-1 had a positive effect on breast cancer cell cycle arrest in the G0/G1 phase. These results indicated that miR-1 triggered the cycle arrest in G1/G0 phase, resulting in the inhibition of breast cancer cell growth.

Taken together, these findings revealed that miR-1 could function as a gastric and breast cancer suppressor by inducing cell cycle arrest.

### Mechanism of miR-1-mediated inhibition of gastric and breast cancer cell growth

**Figure 3:**
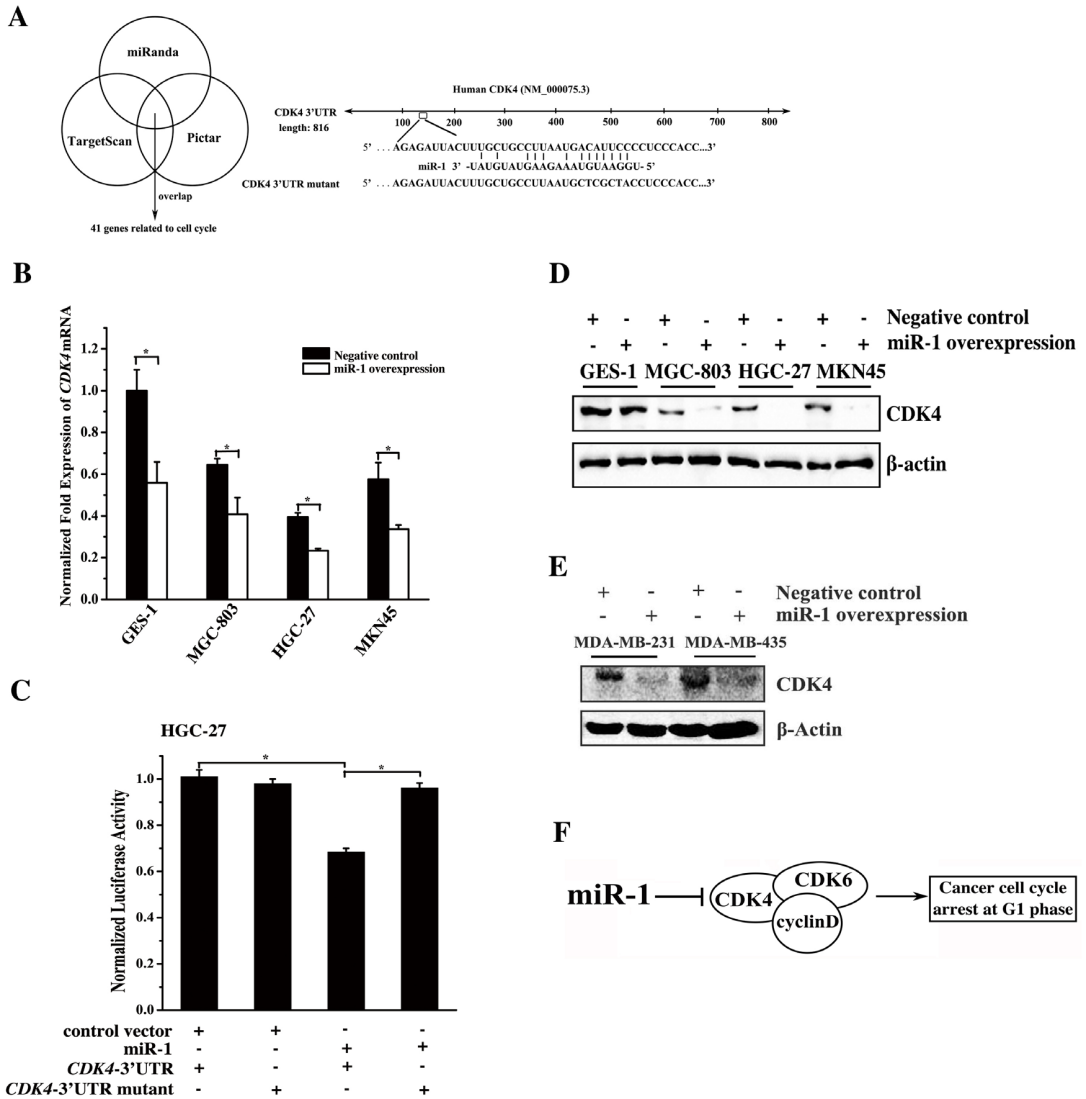
Mechanism of gastric and breast cancer cell cycle regulation mediated by miR-1 **A**. Prediction of miR-1 target genes. The miR-1 targets related to the cell cycle were predicted using the miRanda, TargetScan and PicTar algorithms. *CDK4* was predicted to be a target gene of miR-1. **B**. Expression levels of endogenous *CDK4* in response to miR-1 overexpression in cells. Cells were transfected with the miR-1 precursor or the negative control. Twenty hours later, the level of *CDK4* mRNA was detected using quantitative real-time PCR. **C**. Interaction between miR-1 and *CDK4*. The miR-1 construct or the vector only and the luciferase plasmid containing the 3′UTR of *CDK4* mRNA or its mutant were co-transfected into HGC-27 cells. Then the luciferase activities were detected. The *Renilla* luciferase values were normalized to the firefly luciferase values. **D**. Western blot analysis of CDK4. Cells were treated with 30 nM of the miR-1 precursor or the negative control RNA for 48 h and whole-cell extracts were analyzed by Western blot analysis. β-actin was used for protein loading correction. **E**. Effects of miR-1 overexpression on its target gene expression in breast cancer cells. Breast cancer cells were transfected with 30 nM of the miR-1 precursor or the negative control miRNA. After 48 h, the expression of CDK4 in the cells was analyzed by Western blot. β-actin was used as a control. **F**. Model for the miR-1-mediated pathway in the cell cycle of cancer cells. Statistically significant differences are indicated with asterisks (*, *p* < 0.05; ** *p* < 0.01).

To explore the molecular mechanism of miR-1-mediated cell cycle arrest in cancer cells, the miR-1 targets related to the cell cycle were predicted using the miRanda, TargetScan and PicTar algorithms. The results showed that 41 genes including *CDK4* were the potential targets of miR-1 (Figure 3A). To further reveal the genes targeted by miR-1, miR-1 was overexpressed in cells, and the expression levels of the 41 target genes were then detected. The quantitative real-time PCR data revealed that *CDK4* was significantly downregulated compared with the controls when miR-1 was overexpressed (Figure 3B). However, the expression levels of the other 40 potential targets related to the cell cycle showed little change in response to miR-1 overexpression (data not shown). The results indicated that *CDK4*, a key gene required for the G1-S transition in the cell cycle [12], was a target of miR-1. This gene contains an miR-1 recognition site in the 3′UTR of *CDK4* (Figure 3A). To determine whether miR-1 could directly interact with *CDK4*, the *CDK4* 3′UTR and *CDK4* 3′UTR mutant constructs were generated (Figure 3A). Then, cells were co-transfected with the constructs and miR-1. The dual-luciferase reporter assays indicated that the luciferase activity after the treatment with miR-1+ *CDK4* 3′UTR was significantly reduced compared with that of the controls (Figure 3C), indicating that miR-1 bound to the 3′UTR of CDK4. The above data revealed that *CDK4* was a target gene of miR-1.

To assess the effect of miR-1 overexpression on the expression of *CDK4 in vivo*, the miR-1 precursor was transfected into the gastric or breast cancer cells, and then the CDK4 protein level was determined. The Western blot results indicated that miR-1 overexpression led to a significant decrease in the level of CDK4 protein in the examined cells (Figure 3D and 3E), indicating that miR-1 could target *CDK4* in gastric or breast cancer cells.

These results demonstrated that miR-1 could mediate gastric or breast cancer cell cycle arrest in the G0/G1 phase by directly targeting the *CDK4* gene (Figure 3F).

### Effects of miR-1 on gastric and breast cancerous cell metastasis

**Figure 4:**
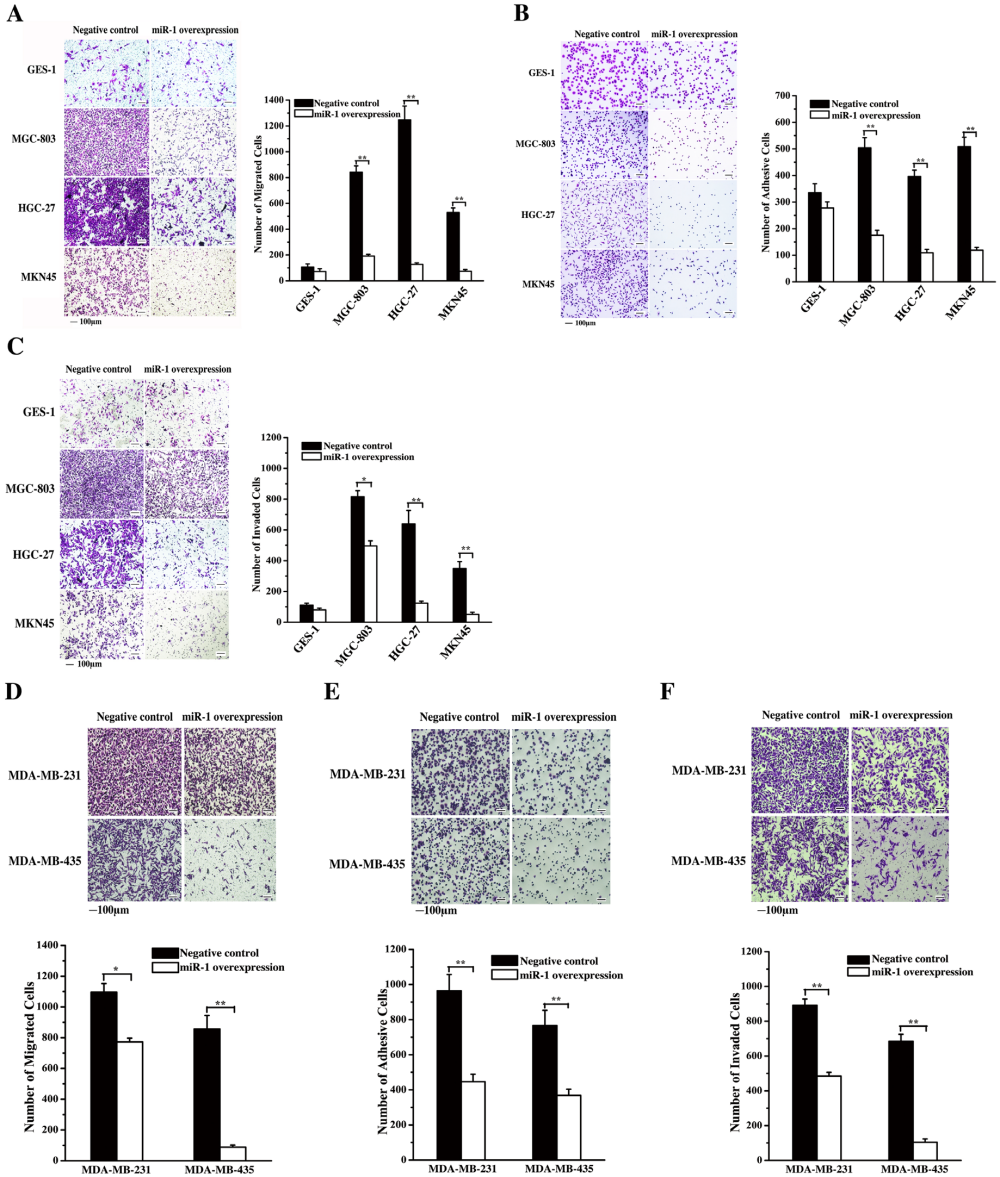
Effects of miR-1 on gastric and breast cancerous cell metastasis **A**. Effect of miR-1 on gastric cancer cell metastasis. Gastric cancer cells (MGC-803, HGC-27 and MKN45) and the normal cells (GES-1) were transfected with the miR-1 precursor or the negative control. At 48 h after transfection, cell migration was examined, and the data were analyzed. Representative images are shown. Scale bar, 100 μm. **B**. Effects of miR-1 overexpression on the adhesion of gastric cancer cells. The cells were transfected with the miR-1 precursor or the negative control and then cultured for 48 h. The number of adherent cells was evaluated. Representative images are shown. Scale bar, 100 μm. **C**. The role of miR-1 in cancer cell invasion. miR-1 precursor-transfected cells were cultured for 48 h. Then, the cells were characterized with cell invasion assays. Scale bar, 100 μm. **D**. Evaluations of breast cancer cell migration. MDA-MB-231 and MDA-MB-435 cells were transfected with 30 nM of the miR-1 precursor or the negative control miRNA to overexpress miR-1. At 48 h after transfection, the migrated cells were examined. Representative images are shown. Scale bar, 100 μm. **E**. The influence of miR-1 overexpression on breast cancer cell invasion. At 48 h after miR-1 overexpression, the invaded cells were evaluated. Representative images are provided. Scale bar, 100 μm. **F**. Cell adhesion assays. Breast cancer cells treated with the miR-1 precursor were cultured for 48 h, then, the adherent cells were examined. Representative images are indicated. Scale bar, 100 μm. In all panels, asterisks represent statistically significant differences between treatments (*, *p* < 0.1; **, *p* < 0.05).

To evaluate the effect of miR-1 on the metastasis of gastric cancer cells, miR-1 was overexpressed in cancer cells, followed by detection of cancer cell migration, adhesion and invasion. The results revealed that the miR-1 overexpression significantly inhibited the migration of gastric cancer cells (MGC-803, HGC-27 and MKN45) but not that of normal cells (GES-1) (Figure 4A), indicating that miR-1 might be required for gastric cancer cell metastasis.

The data from the cell adhesion assays showed that the number of adherent cancer cells (MGC-803, HGC-27 or MKN45) was significantly decreased compared with the control when miR-1 was overexpressed in cancer cells (Figure 4B). However, miR-1 overexpression had no significant effect on the number of adherent normal cells (GES-1) (Figure 4B). These results indicated that miR-1 could inhibit the adhesion of gastric cancer cells. At the same time, the cell invasion assays showed that miR-1 overexpression led to a significant decrease in the invasion of cancer cells (MGC-803, HGC-27 or MKN45), whereas the invasion of normal cells (GES-1) was not affected by miR-1 (Figure 4C). The data revealed that miR-1 could inhibit the metastasis of gastric cancer cells.

The cell migration and invasion assay results indicated that the miR-1 overexpression significantly inhibited the breast cancer cell migration and invasion (Figure 4D and 4E). At the same time, the MDA-MB-231 and MDA-MB-435 breast cancer cells displayed significant decreases in their attachments to fibronectin (Figure 4F). These data showed that miR-1 could inhibit the metastasis of breast cancer cells.

### MiR-1-mediated pathway for cancer cell metastasis

**Figure 5:**
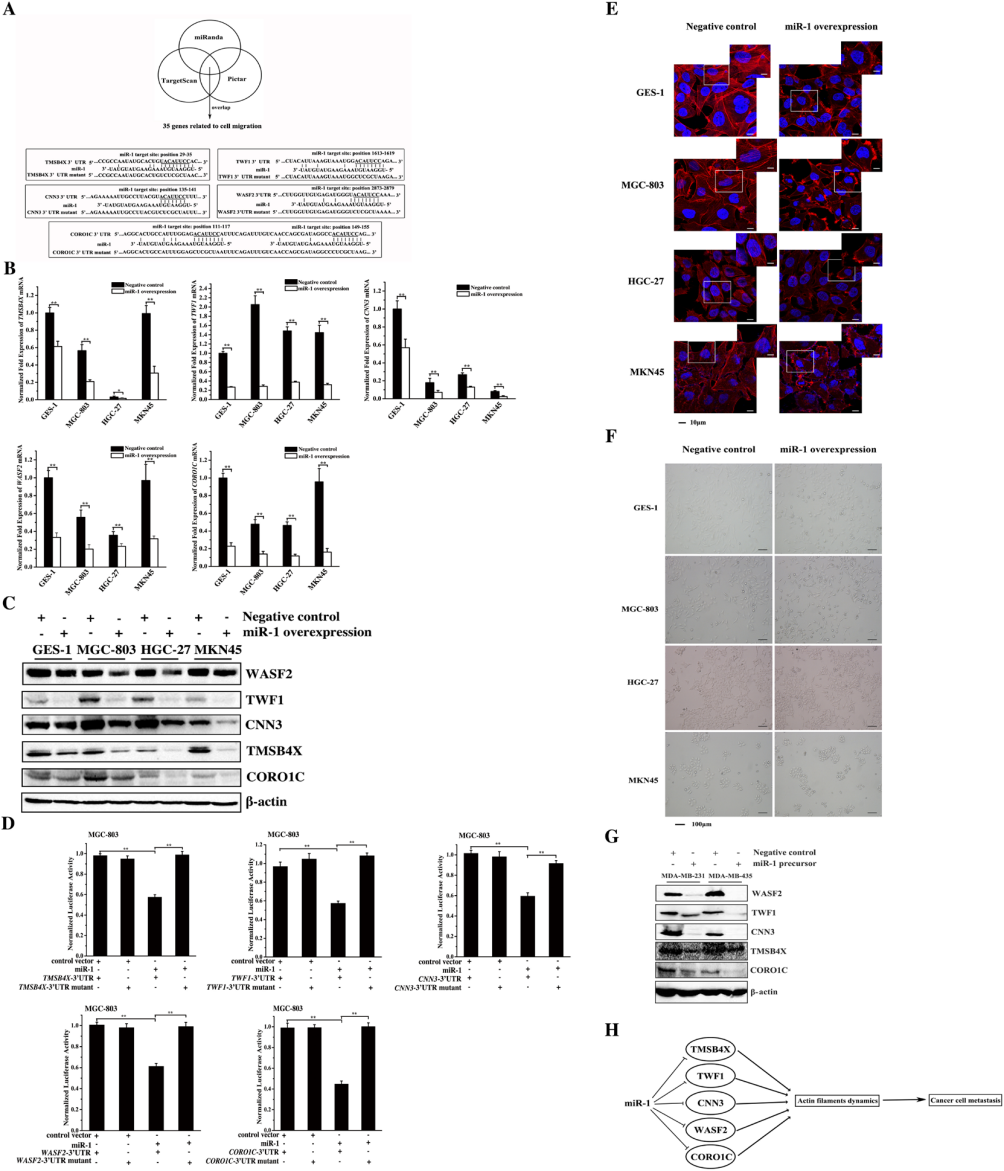
The miR-1-mediated pathway for cancer cell metastasis **A**. The prediction of genes targeted by miR-1. As predicted, 35 genes associated with cell migration were miR-1 targets. The genes (*TMSB4X*, *TWF1*, *CNN3*, *WASF2* and *CORO1C*) targeted by miR-1 are indicated in the boxes. **B**. The interactions between miR-1 and its targets *in vivo*. The miR-1 precursor or the negative control miRNA was transfected into gastric cancer cells (MGC-803, HGC-27 and MKN45) and the normal cells (GES-1). At 24 h after transfection, the mRNAs of the 35 target genes of miR-1 were detected using quantitative real-time PCR. Among the 35 target genes, 5 genes (*TMSB4X*, *TWF1*, *CNN3*, *WASF2* and *CORO1C*) were significantly downregulated in response to miR-1 overexpression. **C**. The influence of miR-1 on target gene expression. Cells were transfected with the miR-1 precursor or the negative control miRNA. At 24 h after transfection, the proteins encoded by the target genes were examined using Western blot analysis. The antibodies used are indicated at the right. **D**. The direct interactions between miR-1 and its target genes. The miR-1 precursor and the 3′ UTR of *TMSB4X*, *TWF1*, *CNN3*, *WASF2* or *CORO1C* were co-transfected into MGC-803 cells. Luciferase activity was measured at 24 h after transfection. And the ratio of firefly luciferase activity to Renilla luciferase activity is shown. **E**. Effects of miR-1 on the actin filament formation. Cells treated with the miR-1 precursor or negative control miRNA for 48h were stained with rhodamine-phalloidin (red) and DAPI (blue) to label F-actin and nuclei, respectively. Then stained cells were subjected to confocal microscopy. Scale bar, 10 μm. **F**. The influence of miR-1 on cell morphology. The morphology of miR-1-overexpressing cells was examined with optical microscopy. Scale bar, 100 μm. **G**. Western blot analysis of WASF2, TWF1, CNN3, TMSB4X and CORO1C. Breast cancer cells were treated with 30 nM of the miR-1 precursor or the negative control miRNA and cultured for 48 h. The whole-cell lysates were subjected to Western blot analysis. β-actin was used as a control. **H**. Model for the miR-1-mediated inhibitory mechanism of cancer cell metastasis. All experiments were biologically repeated three times. The data are shown as the mean ± standard deviation (* *p* < 0.05, ** *p* < 0.01).

The target genes of miR-1 were predicted to elucidate the role of miR-1 in cancer cell metastasis. The results indicated that 35 genes associated with cell migration might be targeted by miR-1 (Figure 5A). To confirm the target gene predictions, miR-1 was overexpressed in gastric cancer cells (MGC-803, HGC-27 and MKN45) and in the normal cells (GES-1). The miR-1 overexpression significantly downregulated 5 genes (*TMSB4X*, *TWF1*, *CNN3*, *WASF2* and *CORO1C*) of the 35 predicted target genes in the examined cells (Figure 5B). Western blot analysis yielded similar results (Figure 5C). These data indicated that *TMSB4X*, *TWF1*, *CNN3*, *WASF2* and *CORO1C* were the target genes of miR-1. To explore the direct interactions between miR-1 and the five target genes, the miR-1 precursor and the 3′ UTR of *TMSB4X*, *TWF1*, *CNN3*, *WASF2* or *CORO1C* were co-transfected into MGC-803 cells. The dual-luciferase reporter assay results indicated that miR-1 significantly reduced the activity of luciferase fused to the 3′ UTR of *TMSB4X*, *TWF1*, *CNN3*, *WASF2* or *CORO1C* compared with the controls (Figure 5D), showing that these 5 genes could be directly targeted by miR-1. These data presented that *TMSB4X*, *TWF1*, *CNN3*, *WASF2* and *CORO1C* genes were the targets if miR-1.

TMSB4X, CORO1C, CNN3, WASF2 and TWF1 are key components required for actin polymerization [13–17]. Therefore the effect of miR-1 on the fiber formation of actin was explored. The confocal microscopy data indicated that the formation of actin stress fibers was destroyed in miR-1-overexpressing cells compared with the control cells (Figure 5E). The miR-1 overexpression resulted in obvious morphological changes in the cells, including a decreased number of adherent cells and an increased number of round cells (Figure 5F).

To investigate the mechanism underlying the miR-1-mediated regulation of breast cancer cell metastasis, the expression levels of *WASF2*, *TWF1*, *CNN3*, *TMSB4X* and *CORO1C* genes in miR-1-overexpressing cells were examined. Western blot data indicated that miR-1 overexpression significantly downregulated WASF2, TWF1, CNN3, TMSB4X and CORO1C (Figure 5G), showing that miR-1 could inhibit breast cancer cell metastasis by targeting the *WASF2*, *TWF1*, *CNN3*, *TMSB4X* and *CORO1C* genes.

Taken together, these data showed that miR-1 could inhibit cancer cell metastasis by targeting actin-associated genes, leading to the inhibition of actin cytoskeleton formation (Figure 5H).

### The underlying mechanism of miR-1-mediated synchronous suppression of tumor growth and metastasis

**Figure 6:**
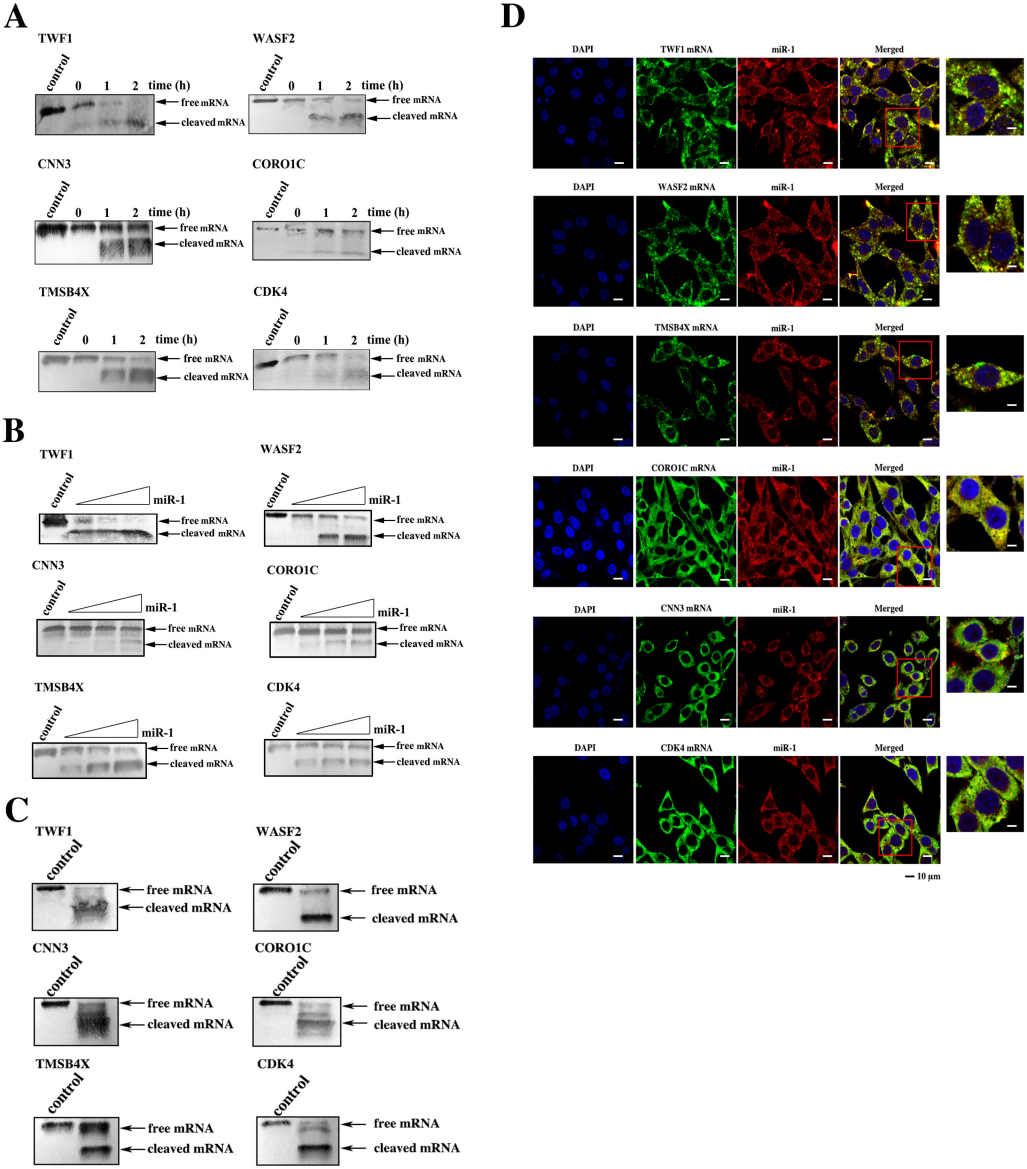
The simultaneous expression regulation of target genes by miR-1 **A**. The time-course assays of the miR-1-guided cleavage of target mRNAs. Each target mRNA of miR-1 was incubated with miR-1 and the Ago2 complex for various times at 37°C. The mRNA alone was used as a control. The cleavage products were examined using Northern blot analysis. The probes are shown at the top, and the numbers indicate the cleavage time. **B**. The effect of miR-1 concentration on target cleavage. Each miR-1 target mRNA was incubated with the Ago2 complex and different concentrations of miR-1 for 1 h. Then the cleavage products were detected by Northern blot analysis. The mRNA alone was used as a control. **C**. The miR-1-guided simultaneous cleavage of miR-1 target mRNAs. The mRNAs of all six miR-1 targets were equivalently co-incubated with the Ago2 complex and miR-1. One hour later, the cleavage products were examined by Northern blot analysis. The mRNA alone was used as a control. **D**. The intracellular co-localization of miR-1 and its six target genes. MDA-MB-435 cells were transfected with the miR-1 precursor and then cultured for 48 h. The cells were subjected to fluorescence *in situ* hybridization using a DIG-labeled miR-1 probe and a biotin-labeled mRNA probe. Confocal microscopy images are presented. Green, target gene mRNA. Red, miR-1. Blue, nuclei. Scale bar, 10 μm.

The above data indicated that miR-1 could inhibit tumor growth and metastasis by targeting *CDK4* gene and *TWF1*, *WASF2*, *TMSB4X*, *CNN3* and *CORO1C* genes, respectively. To explore whether miR-1 could simultaneously suppress tumor growth and metastasis by synchronously regulating the expressions of these six target genes, the miR-1-guided cleavage of the target mRNAs in the Ago complex was investigated. The time-course results showed that all six targets could be cleaved in the miR-1-Ago complex (Figure 6A). The miR-1-guided cleavage of target mRNAs in the Ago complex occurred in a miRNA-concentration-dependent manner (Figure 6B), indicating that miR-1 could guide the cleavage of its targets. When the six target mRNAs were co-incubated at equivalent levels with the miR-1-Ago2 complex, all six mRNAs were cleaved (Figure 6C). These findings revealed that miR-1 could simultaneously guide the cleavage of its six target mRNAs.

To investigate whether miR-1 could synchronously target the mRNAs of its six target genes in cells, the co-localization of miR-1 and its targets was assessed. The confocal microscopy images showed that miR-1 was co-localized with the mRNAs of the six target genes (Figure 6D). Therefore, miR-1 could simultaneously regulate the expressions of the six target genes in cells.

These findings presented that miR-1 simultaneously suppressed tumor growth and metastasis by synchronously targeting multiple genes.

### The miR-1-mediated suppression of tumor growth and metastasis *in vivo*

**Figure 7:**
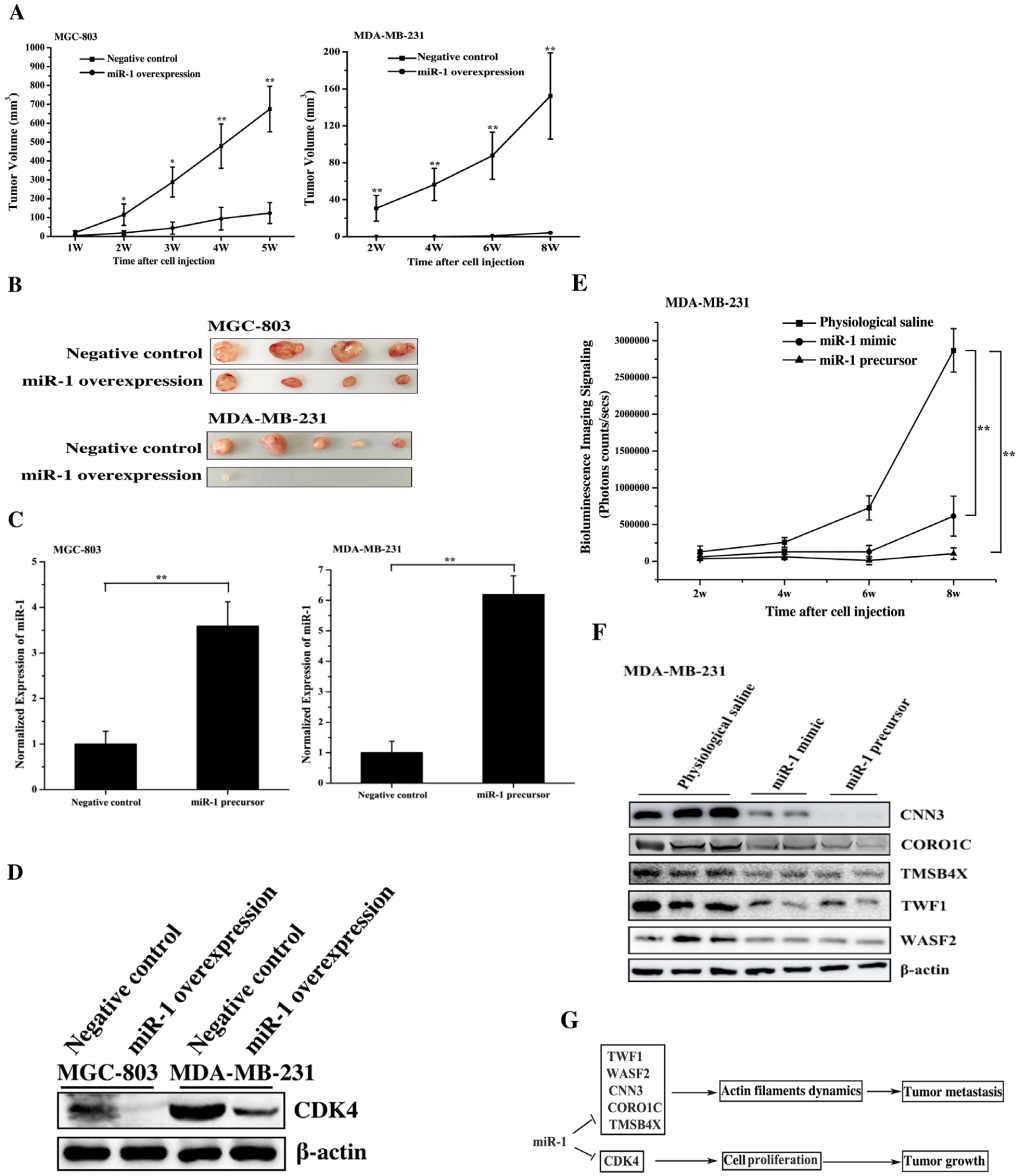
The miR-1-mediated tumor suppression *in vivo* **A**. Effect of miR-1 overexpression on tumor growth *in vivo*. Gastric cancer cells (MGC-803) or breast cancer cells (MDA-MB-231) were transfected with the miR-1 precursor or the negative control. At 48 h after transfection, the cells were subcutaneously injected into nude mice. And subsequent tumor growth was examined weekly. **B**. The sizes of the tumors in mice. Mice were treated with miR-1-overexpressing MGC-803 cells or miR-1-overexpressing MDA-MB-231 cells. As controls, cells transfected with the negative control were also injected into mice. **C**. The miR-1 expression levels in tumors derived from miR-1-overexpressing MGC-803 cells or miR-1-overexpressing MDA-MB-231 cells using quantitative real-time PCR analysis. **D**. Western blot analysis of the miR-1 target CDK4 in tumors. **E**. The time course evaluation of breast cancer metastasis. The mice that received different treatments were subjected to bioluminescence imaging at various time points after the cell injection. **F**. The effect of miR-1 on the expression levels of its target genes in solid tumors. The solid tumors were analyzed by Western blot to detect the expression of WASF2, TWF1, CNN3, CORO1C and TMSB4X. The treatments are indicated at the top. **G**. Model for the miR-1-mediated inhibitory mechanism of tumor growth and metastasis. In all panels, asterisks indicate significant differences (*, *p* < 0.05; **, *p* < 0.01) between treatments.

To explore the role of miR-1 in tumorigenesis *in vivo*, miR-1 was overexpressed in gastric cancer cells (MGC-803) or breast cancer cells (MDA-MB-231), and the cancer cells were then injected into nude mice to examine the tumor growth. The results indicated that the tumor growth was significantly inhibited in the mice treated with miR-1-overexpressing MGC-803 cells or miR-1-overexpressing MDA-MB-231 cells compared with the control cells (Figure 7A). The tumor sizes of mice injected with miR-1-overexpressing MGC-803 or MDA-MB-231 cells were significantly larger than those of the controls (Figure 7B). These data indicated that miR-1 could inhibit the growth of gastric and breast cancer tumor *in vivo*.

Quantitative real-time PCR analysis indicated that the expression level of miR-1 in tumors was 3.5-6 times higher than that in the controls (Figure 7C), showing the upregulation of miR-1 in gastric and breast cancers *in vivo*. The western blot data indicated that CDK4 protein was significantly downregulated in tumors derived from miR-1 overexpressing cancer cells compared with that in the controls (Figure 7D). These results showed that miR-1 inhibited the growth of gastric and breast cancer by downregulating the expression of its target, CDK4, *in vivo*.

To investigate the influence of miR-1 on cancer metastasis *in vivo*, the animal model of breast cancer cell metastasis to lung was established according to the published protocols [6, 13, 19]. MDA-MB-231 cells expressing luciferase were transfected with the miR-1 precursor, and these cells were then intravenously injected into mice via the tail vein. At the same time, the synthesized miR-1 (miR-1 mimic) or physiological saline was injected into the mice treated with MDA-MB-231 cells. The data revealed that metastasis of the breast cancer cells to the lungs of the mice was significantly suppressed by miR-1 mimic or miR-1 precursor compared with the negative control (Figure 7E), indicating that miR-1 could inhibit tumor metastasis. The time course assays showed that the inhibition of breast cancer metastasis appeared 3 weeks after the cell injection (Figure 7E). These findings demonstrated that miR-1 could inhibit breast cancer metastasis *in vivo*.

Western blot analysis of metastatic solid tumors indicated that the expression levels of miR-1 targets including WASF2, TWF1, CNN3, CORO1C and TMSB4X were significantly downregulated in the solid tumors of miR-1-overexpressing mice compared with the control mice (Figure 7F). Therefore, miR-1 could suppress cancer metastasis *in vivo* by downregulating target gene expression.

Taken together, these findings showed that miR-1 could efficiently inhibit tumorigenesis and metastasis *in vivo* by targeting CDK4, WASF2, TWF1, CNN3, CORO1C and TMSB4X (Figure 7G).

## DISCUSSION

The development of cancer is a complex multi-step process. To date, many studies have focused on this process to inhibit tumor growth and to block cancer cell metastatic dissemination. MiRNAs, as oncogenes or tumor suppressors, play negative or positive roles in cancer development by inhibiting the expressions of genes related to tumorigenesis or metastatic dissemination [24]. The aim of our study was to explore the underlying mechanism of miRNA-mediated simultaneous suppression of both tumor growth and metastasis during cancer development. In the present investigation, miR-1 was shown to simultaneously inhibit tumor growth and metastasis of breast and gastric cancers by synchronously targeting *CDK4* and *TMSB4X*, *CNN3*, *TWF1*, *CORO1C* and *WASF2* genes. Therefore, our study identified a novel mechanism of simultaneous inhibition of tumor growth and metastasis mediated by a miRNA (miR-1).

As well known, CDK4 is required for the cell cycle transition from the G1 phase into the S phase. The downregulation of *CDK4* expression mediated by miR-1 caused tumor cell cycle arrest in the G0/G1 phase, resulting in inhibited cancer cell proliferation. On the other hand, the findings of this study revealed that *TMSB4X*, *CNN3*, *TWF1*, *CORO1C* and *WASF2* genes were directly targeted by miR-1. The *TMSB4X*, *CNN3*, *TWF1*, *CORO1C* and *WASF2* genes encode cytoskeletal proteins related to actin filament dynamic regulation, and they are involved in cancer metastasis [22, 25–35]. The human breast tumor biomarker miR-30c can inhibit tumor invasion by targeting the cytoskeletal network genes encoding TWF1 and vimentin [25]. As previously reported, miR-146a suppressed the migration and invasion of gastric cancer cells by targeting WASF2 [29]. In colorectal cancer, the expression of CNN3 is significantly increased and is a marker for the detection of lymph node metastasis [31]. It is reported that miR-206 represses the migration of breast cancer cells via the post-transcriptional regulation of CORO1C [33]. Recent studies have shown that TMSB4X promotes metastasis in malignant tumors. In this context, the miR-1-mediated downregulation of the *TMSB4X*, *CNN3*, *TWF1*, *CORO1C* and *WASF2* genes led to the inhibition of tumor cell metastasis of breast and gastric cancers.

Generally, a single miRNA can target multiple genes [14]. Thus, miRNAs can influence tumorigenesis or tumor metastasis by inhibiting the expression of one or several targets. For example, miRNA-122 inhibits the tumorigenic properties of hepatocellular carcinoma cells by repressing ADAM10 (a disintegrin and metalloprotease family 10), SRF (serum response factor), and Igf1R (insulin-like growth factor 1 receptor), which promote tumorigenesis [37]. However, no direct evidence has shown that miR-122 simultaneously interacts with its targets in cells. To date, whether a single miRNA synchronously targeted multiple genes in cells has not been addressed. In the present study, we showed that miR-1 could simultaneously cleave six targets *in vitro* and that miR-1 was co-localized with its targets in cells. Therefore, our study presented direct evidence that a single miRNA could simultaneously target various genes indeed.

In this investigation, miR-1 was shown to be significantly downregulated in gastric and breast cancer cells compared with normal cells. Previous studies have shown that miR-1 expression is decreased in several types of cancers, such as prostate cancer [38–40], lung cancer [41], bladder cancer [42, 43], hepatocellular carcinogenesis [44], head and neck squamous cell carcinoma [45] and thyroid carcinogenesis [46]. Our results are consistent with these previous findings. Based on the results of our studies and those of previous studies, miR-1 could serve as a biomarker for the clinical diagnosis of tumors and the restoration of miR-1 in cancers might be a potential therapeutic strategy for cancer. These issues merit further investigation.

## MATERIALS AND METHODS

### Cell culture

Human gastric cancer cell lines (MGC-803, MKN45, MKN74 and AGS) and a human epithelium-derived, normal gastric cell line (GES-1) were cultured in RPMI 1640 medium (Gibco, USA) supplemented with 10% fetal bovine serum (FBS). Human gastric cancer HGC-27 cells, human skin cancer A375 cells, human normal skin CCC-ESF cells and human MCF-7 breast cancer cells were maintained in DMEM (Gibco, USA) supplemented with 10% FBS. Human breast cancer cells, including MDA-MB-435, MDA-MB-468 and MDA-MB-231 cells, were cultured in Leibovitz's L-15 medium (Gibco, USA) supplemented with 10% FBS. Normal human MCF-10A breast cells were cultured in DMEM/F12 (Gibco, USA) supplemented with 5% horse serum, 10 μg/ml insulin, 20 ng/ml EGF, 100 ng/ml cholera toxin and 0.5 μg/ml hydrocortisone. All cells were cultured in a humidified atmosphere of 5% CO_2_ and 95% air at 37°C. All cells were purchased from The Type Culture Collection of The Chinese Academy of Sciences, Shanghai, China.

### Quantitative real-time PCR for the quantification of miR-1

To detect the expression of miR-1, total RNA was extracted from cells and tissues with a mirVana miRNA Isolation Kit (Ambion, USA). After treatment with DNase I, the cDNA was reverse transcribed from the total RNA using an miRNA-specific primer with a TaqMan® microRNA reverse transcription kit (Applied Biosystems, USA). The real-time PCR reaction mixture (10 μl) contained 0.5 μl of RT product, 5 μl of TaqMan 2×Universal PCR Master Mix (Applied Biosystems) and 1μl of TaqMan miRNA Assay reagent (Applied Biosystems). Real-time PCR was performed at 95°C for 10 min, followed by 50 cycles at 95°C for 15s and 60°C for 1 min. The 2^−(ΔΔCt)^ method [42] was used to determine relative individual miRNA quantities, and U6 (Applied Biosystems) was used as an internal standard for normalization. The assays were performed in triplicate.

### Pathological analysis of gastric cancer patients

After the gastric cancer patients were examined by gastroscopy, paired normal and cancerous tissue samples were resected from each patient. Tissue samples were fixed in 10% buffered formalin for 12 h; and were then washed with phosphate-buffered saline (PBS). Subsequently, the samples were transferred to 70% ethanol, embedded in paraffin and sectioned. After being stained with hematoxylin and eosin, the samples were examined with white light microscopy and polarized light microscopy.

### Gastric sample preparation

The resected cancerous tissues and adjacent normal tissues were immediately immersed in RNAlater solution (Ambion, USA), kept overnight, and then stored at -80°C for later use.

### Overexpression of miR-1 in cells

Cells were transfected with 30 nM of the miR-1 precursor (Ambion, USA) or the negative control miRNA (Ambion, USA) using Lipofectamine® RNAiMAX (Life Technologies, USA). At 48 h after transfection, total RNA was extracted with a mirVana miRNA Isolation Kit (Ambion, USA), followed by treatment with DNase I (Tiangen, Shanghai, China). The cDNAs were reverse transcribed from the total RNA using an miRNA-specific primer with a TaqMan® microRNA reverse transcription kit (Applied Biosystems, USA). Then, real-time PCR was conducted at 95°C for 10 min, followed by 50 cycles at 95°C for 15s and 60°C for 1 min. The real-time PCR reaction mixture (10 μl) contained 0.5 μl of reverse transcription product, 5 μl of TaqMan 2×Universal PCR Master Mix (Applied Biosystems, USA), and 1μl of TaqMan miRNA Assay reagent (Applied Biosystems, USA). The expression of miR-1 was normalized to U6 (Applied Biosystems, USA). All reactions were performed in triplicate.

### Cell proliferation assay

Cells were plated in 96-well plates, incubated overnight, and then transfected with 30 nM of the miR-1 precursor or the negative control miRNA (Ambion, USA) using Lipofectamine® RNAiMAX (Life Technologies, USA). Cell proliferation was monitored every 24 h for 5 days using a CellTiter 96® AQueous One Solution Cell Proliferation Assay Kit (Promega, USA) according to the manufacturer's protocol. To measure cell proliferation, 20 μl of CellTiter 96® AQueous One Solution Reagent was added to each well, and the plates were incubated at 37°C for 3 h. Absorbance was measured at 490 nm using the iMARK^TM^ microplate reader (Bio-Rad, USA).

### Cell cycle assay

Fluorescence-activated cell sorting (FACS) analysis was used to examine the cell cycle of gastric cancer and normal cells. The cancer cells (MGC-803, HGC-27 and MKN45) and normal cells (GES-1) were transfected with 30 nM of the miR-1 precursor or the negative control miRNA (Ambion, USA). At 48 h after transfection, the cells were collected and fixed with 70% ethanol overnight at 4°C. Then, the cells were centrifuged at 300× *g* for 10 min. After being washed with PBS, the cells were stained with propidium iodide solution [20 μg/ml of propidium iodide (Sigma, USA), and, 200 μg/mL of RNaseA (Sangon, China)] for 30 min at 37°C in the dark. Subsequently the cells were examined using FACS-Calibur flow cytometer (BD Biosciences, USA). Flow cytometric data were analyzed using Cell Quest Pro software (BD Biosciences, USA).

### Detection of cell senescence

Senescence was analyzed using a senescence β-galactosidase staining kit (Beyotime Institute of Biotechnology, Shanghai, China). Cells were transfected with the miR-1 precursor or the negative control miRNA. At 48 h after transfection, the cells were collected and fixed with fixing solution (Beyotime, China) for 15 min. The cells were stained with senescence-associated β-galactosidase staining solution (Beyotime, China) overnight at 37°C.

### Western blot analysis

Cells transfected with the precursor miR-1 or the negative control miRNA were collected by centrifugation at 300× *g* for 10 min and were then lysed using radio immunoprecipitation assay buffer (Beyotime, China) containing 2 mM phenylmethanesulfonyl fluoride (PMSF). The proteins were separated by SDS-PAGE and electrotransferred to polyvinylidene fluoride membranes (Millipore, USA). After being incubated in blocking solution [4% bovine serum albumin (BSA) in TBST (Tris-buffered saline and Tween-20)] for 2 h at temperature, the membranes were incubated with primary antibodies overnight at 4°C. Subsequently, the membranes were incubated with AP-conjugated secondary antibodies (Roche, USA) for 2 h at room temperature. After being washed with TBST, the membranes were then incubated with BCIP/NBT substrate (Sangon, China) until the blots were visualized. The antibodies used in this study were purchased (Abcam, USA).

### Apoptosis assay

Cells transfected with the miR-1 precursor or the negative control miRNA were collected 48 h after transfection by centrifugation at 300 × *g* for 10 min. After being washed with cold PBS, the cells were stained with FITC-AnnexinV and propidium iodide using a FITC Annexin V apoptosis detection kit (BD Biosciences, USA) according to the manufacturer's recommendations and were immediately analyzed by flow cytometry (BD Biosciences, USA). Flow cytometric data were analyzed using Cell QuestPro software (BD Biosciences, USA). Cells were divided into viable cells, dead cells, early apoptotic cells, and apoptotic cells and the percentages of apoptotic cells were calculated. All the experiments were conducted in triplicate.

### Prediction of miR-1 target genes

The putative target genes of miR-1 were predicted using the miRanda, TargetScan and PicTar algorithms (Creighton et al, 2008; Doran and Strauss, 2007; Krek et al, 2005). The overlapping target genes were further investigated.

### Quantitative real-time PCR analysis of mRNA

Cells were transfected with 30 nM of the miR-1 precursor or the negative control for miR-1 (Ambion, USA). At 24h after transfection, the cells were collected, and total RNA was extracted with an RNAprep pure Tissue Kit (Tiangen, China). First-strand cDNA was synthesized by reverse transcription with a PrimeScript 1st Strand cDNA Synthesis Kit (TaKaRa, Japan). Quantitative real-time PCR was conducted with gene-specific primers. The reaction mix consisted of 10 μl of SYBR Green PCR Master Mix (TaKaRa, Japan), 0.4 μl of 10μM forward and reverse primers and 0.5 μl of cDNA in a final volume of 20 μl. The PCR conditions were 30 s at 95°C, followed by 40 cycles of 95°C for 5 s and 60°C for 30 s. The expression levels of genes were normalized to glyceraldehyde-3-phosphate dehydrogenase (GAPDH).

### Dual-luciferase reporter assay

The 3′UTR of *CDK4*, *TMSB4X*, *TWF1*, *CNN3*, *WASF2* or *CORO1C* was amplified with sequence-specific primers (CDK4, 5′-CATTTC CCTTCTGGACACT G-3′ and 5′-GCCTCAGTCTCCCAAGTAGC-3′; TMS B4X, 5′-TGCGCCGCCAAT ATGCACTGTAC-3′ and 5′-CCTGCCAGCCAGATAGATAGAC-3′; TWF1, 5′-TT CCATGGGTGTACACGTAGA-3′ and 5′-ATGTCCAC CAGAAGGCATGTAA-3′; CNN3, 5′-ATCCACACAG AAGGAGCTCAGT-3′ and 5′-CAAATGCATCACCCA GGCCTA-3′; WASF2, 5′-CTTTAGACCCAGAGCC CTTTAAGA-3′ and 5′-AGAG ACCTCAATCTGT CCAAGCT-3′; CORO1C, 5′-AGCTGGTTATTGGTG TGGTCC TA-3′ and 5′-ATGAGAGCGGTGGTAATATGAATC-3′). Then, it was cloned into the pmirGLO dual-luciferase miRNA target expression vector (Promega, USA). As a control, the seed sequence of 3′ UTR of the *CDK4*, *TMSB4X*, *TWF1*, *CNN3*, *WASF2* or *CORO1C* was randomly mutated and cloned into pmirGLO dual-luciferase miRNA target expression vector, generating *CDK4*-3′UTR-mutant, *TMSB4X*-3′UTR-mutant, *TWF1*-3′UTR-mutant, *CNN3*-3′UTR-mutant, *WASF2*-3′UTR-mutant or *CORO1C*-3′UTR-mutant, respectively. The primers used for the amplifications of the 3′-UTR mutants were sequence-specific (*CDK4*, 5′-AGAGGTGGGAGGACATTCCCATTA AGGCAGC-3′ and 5′-GCTGCCTTAATGGGAATGTCCTCCCACCTCT-3′; TMSB4X, 5′-CCAATATGCACTGTCTCGCTAACAAGCATTGC-3′ and 5′-GAAG GCAATGCTTGTTAGCGAGACAGTGCATAT-3′; TWF1, 5′-GTAAATGGCTCG CTAAGAATATAGATGTGATTA-3′ and 5′-CATCTATA TTCTTAGCGAGCCATT TACTTTAATGT-3′; CNN3, 5′-TTGCCTTACGTCTCGCTATTTTTCCTTTTTCTG -3′ and 5′-AAGGAAAAATAGCGAGACGTAAGGCAATTTTTC-3′; WASF2, 5′-TGAGATGGGTCTCGCTAAAAGGAGCAGCCT-3′ and 5′-CTGCTCCTTTTAG CGAGACCCATCTCACAAC-3′; CORO1C, 5′-CCATTTG GAGCTCGCTAATTTC AGATTTGTC-3′ and 5′-AATCTGAAATTAGCGAGCTCCAAATGGCAG-3′).

For the dual-luciferase reporter assays, HGC-27 cells were plated in a 96-well plate, incubated overnight, and then co-transfected with the miR-1 miExpress vector (Applied Biosystems, USA) or the vector only and *CDK4*-3′UTR, *TMSB4X*-3′UTR, *TWF1*-3′UTR, *CNN3*-3′UTR, *WASF2*-3′UTR or *CORO1C*-3′UTR constructs using Attractene Transfection Reagent (Qiagen, USA). As controls, *CDK4*-3′UTR-mutant, *TMSB4X*-3′UTR-mutant, *TWF1*-3′UTR-mutant, *CNN3*-3′UTR-mutant, *WASF2*-3′UTR-mutant and *CORO1C*-3′UTR-mutant were included in the co-transfections. At 24 h after transfection, the cells were collected and washed twice with PBS. Subsequently, passive lysis buffer (Promega, USA) was added to promote the rapid lysis of cells. After incubation for 15 min at room temperature, the lysate was used to detect luciferase activity. The luciferase activity detection was performed according to the protocol for the Dual-Luciferase Assay Reagent (Promega, USA). All the experiments were repeated for three times.

### Cell migration assay

Cell migration was assessed using 24-well Boyden chambers (Costar, Corning, USA) with 8-μm -inserts. Cells were transfected with the miR-1 precursor or the negative control miRNA and were then incubated for 48 h. Then, the cells were trypsinized, and 5 × 10^4^ cells were resuspended in 100 μl serum-free media and added to the inserts. The inserts were placed in wells with 600 μl of 10% serum-containing media. After 24 h of incubation, the membranes of the inserts were carefully washed with cold PBS and fixed with 4% paraformaldehyde (Sigma, USA) for 15 min, followed by staining with crystal violet (0.005%, Beyotime, China) for 15 min. Then the cells on the upper sides of the membranes were wiped away with cotton swabs. Images of the migrated cells were collected using a Nikon Ti-S microscope (Nikon, USA). The data are shown as the numbers of migrated cells per 200× field of view for each sample well. All experiments were independently repeated three times.

### Confocal microscopy

Cells were plated in glass bottomed dishes (*In Vitro* Scientific, USA), incubated overnight, and then transfected with 30 nM of the miR-1 precursor or the negative control RNA for 48 h. The transfected cells were fixed in 4% paraformaldehyde for 15 min. After two washes with PBS, the cells were treated with 0.25% Triton X-100 for 15 min, followed by staining with fluorescent phalloidin (R415, Invitrogen, USA) for 40 min and then DAPI (Sigma, USA) for 5 min. Images of F-actin were captured using a Carl Zeiss LSM710 system (Carl Zeiss, Germany).

### Cell adhesion assay

The ability of cells to adhere to fibronectin were also assessed. Cells were seeded in 24-well plates pre-coated with 20 μg/ml fibronectin (Sigma, USA) and incubated at 4°C overnight. Then the plates were washed with PBS twice and were incubated with 10% BSA at 37°C to block non-specific binding proteins. Cells (5×10^4^) treated with 30 nM of the miR-1 precursor or the negative control were resuspended in serum-free medium and transferred to the coated plates. Subsequently, the cells were allowed to adhere for 30 min at 37°C. To remove non-adherent cells, the plates were washed with PBS twice. The adherent cells were then fixed with 4% paraformaldehyde and stained with a methylrosanilnium chloride solution (Beyotime, China) for 15 min, and examined under a microscope.

### Cell invasion assay

Cell invasion was assessed using 24-well Matrigel invasion chambers (Costar, Corning, USA) with 8-μm-inserts. Cells were transfected with 30 nM of the miR-1 precursor or the negative control. Forty-eight hours later, the cells were trypsinized. Subsequently, the cells were resuspended in 100 μl of serum-free media and added to the inserts, which were placed into wells with 600 μl of 10% serum-containing media. After 24 h of incubation, the membranes of the inserts were carefully washed with cold PBS, fixed with 4% paraformaldehyde (Sigma, USA) for 15 min, and stained with crystal violet (0.005%, Beyotime, China) for 15 min. The cells on the upper side of the membrane were removed with a cotton swab. Images of invaded cells were collected using a Nikon Ti-S microscope (Nikon, USA).

### miR-1-guided cleavage of miR-1 target mRNA

A 500 -bp fragment of miR-1 target's 3′-UTR was amplified with sequence-specific primers (TWF1, 5′-GATCACTAATACGACTCACTATAGG GTGCATTA TCAGTTACAACCT-3′ and 5′-TGGCAC TCTGATTAAACTGCAT-3′; WASF2, 5′- GATCACTAATACGACTCACTATAGGGCTTTAGACCCAGAGCCCT TTAAGA-3′ and 5′-AGAGACCTCAATCTGTCCAA GCT-3′; TMSB4X, 5′-GATCACTAAT ACGACTCACTATAGGGTGCGCCGCCAATATGCACTGT-3′ and 5′-TGGCACT CTGATTAAACTGCAT-3′; CNN3, 5′- GATCACTAATACGACTCACTATAGG GATCCACA CAGAAGGAGCTCAGT-3′ and 5′-CAAATGCATCA CCCAGGCCT A-3′; CORO1C, 5′- GATCACTAATA CGACTCACTATAGGGAGCTGGTTATT GGTGTG GTCCTA-3′ and 5′-ATGAGAGCGGTGGTAATA TGAATC-3′; CDK4, 5′- GATCACTAATACGACTCACTA TAGGGCATGGAAGGAAGAAAAGCTG-3′ and 5′-TTC AAGCGATCCTCCTGCCT-3′). Then, the PCR products were used as templates for target mRNA transcription *in vitro* with a T7 transcription kit (TaKaRa, Japan). *In vitro* transcription was performed according to the manufacturer's protocol.

To facilitate the miR-1-guided cleavage of target mRNA, the endogenous Ago2 complex was obtained. The cultured cells were lysed with lysis buffer (20 mM Tris-HCl, 150 mM NaCl, 1.5 mM MgCl_2_, 0.25% NP-40, 1 mM PMSF, pH7.5) for 15 min on ice and then centrifuged at 10,000 × *g* for 10 min at 4°C. The cell lysate was incubated with Ago2 antibody for 1 h at 4°C. Subsequently protein-G-coupled agarose beads were equilibrated with lysis buffer and incubated with the cell lysate and Ago2 antibody for 4 h at 4°C. Then, the beads were washed three times with wash buffer (50 mM Tris-HCl, 300 mM NaCl, 5 mM MgCl_2_, 0.1% NP-40, pH 7.5) and suspended in cleavage reaction buffer (100 mM KOAc, 40 mM HEPES, 5 mM MgCl_2_, 2 mM DTT, 0.35% Triton X-100, 1 mM phenylmethanesulfonyl fluoride, pH 7.6).

The target mRNA (200 ng) was incubated in 20 μl of reaction solution containing 10 μl of Ago2 complex, 2 μl of 10 mM ATP/2 mM GTP solution, and 10 U/ml of RNasin (Promega, USA) and miR-1 (Genepharm, China) at 37°C. At different times after this incubation, the RNA was separated by 1% agarose gel electrophoresis and then transferred to a nylon membrane (GE Healthcare, USA). The RNA was detected using a DIG (digoxigenin)-labeled probe (TWF1, 5′-TCACCATCTAGGTATGA TACTGCCAACTAA-3′; WASF1, 5′-GTTTCTGAGGGCGCTCGGGCTTCCACTG GA-3′; TMSB4X, 5′-TCCTTCCCTGCCAGCCAGATAGATAGACAG-3′; CNN3, 5′-GCAATAAGCATGAGTTTAGTCTTCCATGTAG-3′; CORO1C, 5′-GGAGCC AGCACCATGTGGCTACTGCTTTGA-3′; CDK4, 5′-TCCAAATCGCACAATGGC AAAGCCAAACAG-3′).

### Intracellular co-localization of miR-1 and its target mRNAs

Fluorescence *in situ* hybridization was conducted to intracellularly localize miR-1 and its target mRNAs. MDA-MB-435 cells were transfected with 30 nM of the miR-1 precursor and then cultured for 48 h. The cells were fixed with 4% polyformaldehyde for 15 min at room temperature. After two washes with PBS, the fixed cells were dehydrated in 70% ethanol overnight at 4°C To label an mRNA, the cells were incubated with hybridization buffer [1× SSC (15 mM sodium citrate plus, 150 mM sodium chloride, pH 7.5), 25% (w/v) formamide, 10% (w/v) dextran sulfate, 1× Denhardt's solution] containing 100 nM biotin-labeled probe for 5 h at 37°C. The following probes were used: TWF1 probe (5′-biotin-TGCTCGGAAGTGATAAA GAACA-3′), TMSB4X probe (5′-biotin-TTCTTCCTTCACCAACATGCAA-3′), WASF2 probe (5′-biotin-ACTGAGCTAATGATCTAATCCT-3′), CNN3 probe (5′-fluorescein-AAGAACTGGCTGTACAAGAGTA-3′), CORO1C probe (5′-fluorescein-AGGAC CACACCAATAACCAGCT-3′) and CDK4 probe (5′-fluorescein-TACAGCCAACA CTCCACATGT-3′). The cells were washed with 4× SSC for 10 min, 2× SSC for 5 min and 1× SSC for 5 min. To label miR-1, the cells were incubated with hybridization buffer containing 100 nM DIG-labeled miR-1 probe (5′-DIG-ATACATACTTCTTTACATTCCA -3′) overnight at 37°C. After being washed with an SSC series (4× SSC, 2× SSC and 1× SSC for 10, 5 and 5 min, respectively), the cells were treated with blocking reagent (PBST, PBS containing 1% BSA and 0.02% Tween-20) for 1 h at room temperature, followed by an incubation with 0.3% H_2_O_2_ for 20 min. Subsequently the cells were incubated with horseradish peroxidase (HRP) -labeled anti-DIG antibody (Roche, Switzerland) for 1 h at room temperature and washed with PBS three times. Amplification buffer/H_2_O_2_ stock solution containing Alexa Fluor 546 tyramide (Roche, Switzerland) was added to the cells and incubated for 10 min at room temperature. After washing with PBS, the cells were incubated with HRP-labeled streptavidin (Roche, Switzerland) for 1 h at room temperature, followed by a treatment with 0.3% H_2_O_2_ for 20 min. The cells were washed three times with PBS, and then then amplification buffer/H_2_O_2_ stock solution containing Alexa Fluor 488 tyramide (Roche, Switzerland) were added. After incubating for 10 min, the cells were washed with PBS three times. Finally, the cells were labeled with DAPI (50 ng/ml) (Sigma, USA) for 5 min and washed with PBS. Cell images were captured using a CarlZeiss LSM710 system (Carl Zeiss, Germany).

### Tumorigenicity in nude mice

To evaluate the influence of miR-1 on cancer cell growth *in vivo*, the gastric cancer cells (MGC-803) or the breast cancer cells (MDA-MB-231) were transfected with 30 nM of the miR-1 precursor or the scrambled miRNA (negative control) when the cell density reached approximately 50%. At 48 h after transfection, cells (1×10^6^) were subcutaneously injected into non-obese diabetic severe combined immunodeficiency (NOD/SCID) mice to induce tumor growth. The tumor volumes were measured weekly. Five weeks (MGC-803) or eight weeks later (MDA-MB-231), the NOD/SCID nude mice were euthanized and their tumor sizes were evaluated.

To examine the effects of miR-1 on cancer cell metastasis *in vivo*, the animal model of breast cancer cell metastasis to lung was established as described previously with some modifications [6, 13, 19]. Luciferase expressing MDA-MB-231 cells were constructed by lentiviral packaging to analyze tumor metastasis *in vivo*. Then, MDA-MB-231 cells were transfected with 30 nM of the miR-1 precursor. At 48 h after transfection, cells (2×10^5^) transfected with the miR-1 precursor or control cells (2×10^5^) were intravenously injected via the tail vein into nude mice. Every 3 days after the injection, the mice were intravenously injected via the tail vein with 200 nM of synthesized miR-1 or physiological saline. The metastatic tumors were imaged weekly using an IVIS Spectrum CT Preclinical *In Vivo* Imaging System (PerkinElmer, USA). At 8 weeks after the cell injection, the mice were euthanized and the tumors in the lungs were excised for later use. The animal experiments were approved by The Animal Experiment Center of Zhejiang University, China.

## Abbreviations

TWF1: twinfilin actin binding protein 1
TMSB4X: thymosin beta 4, X-linked
WASF2: WAS protein family member 2
CNN3: calponin 3
CORO1C: coronin 1C
